# Geographic distribution of inflammatory bowel disease in the UK: A spatially explicit survey

**DOI:** 10.1371/journal.pone.0329317

**Published:** 2025-08-28

**Authors:** Mehmet A. Veral, Roger W. Pickup, Manoj Roy, Jeremy Sanderson, Gaurav Agrawal, Peter M. Atkinson

**Affiliations:** 1 Lancaster Environment Centre, Lancaster University, Bailrigg, Lancaster, United Kingdom; 2 Biomedical and Life Sciences Division, Lancaster University, Bailrigg, Lancaster, United Kingdom; 3 Department of Gastroenterology, Guy’s and St Thomas’ NHS Foundation Trust, St Thomas’ Hospital, London, United Kingdom; SKUMS: Shahrekord University of Medical Science, IRAN, ISLAMIC REPUBLIC OF

## Abstract

Inflammatory bowel disease (IBD) is characterized by chronic inflammation in the gastrointestinal (GI) tract, with two main forms: Crohn’s disease (CD) and ulcerative colitis (UC). While CD can affect any part of the digestive system, UC predominately affects the colon and rectum. The incidence and prevalence rates of IBD cases are increasing worldwide, including in Europe where the UK has one of the highest incidence and prevalence rates. This study reports on a new survey of IBD cases in the UK, involving 5,452 respondents. The survey was promoted periodically by multiple IBD organizations across the UK over 307 days (01 Dec 2021–03 Oct 2022) and collected data on participants’ IBD diagnoses and histories. The distributions of CD and UC cases were examined on a grid scale and based on these distributions, relative risk was calculated and mapped in regions where CD and UC cases were recorded. In addition, age- and sex-standardized morbidity rates (ASMRs) for CD and UC were calculated. The results of this UK-wide IBD study reveal an even geographical distribution of reported IBD cases and relative risk across the UK. The ASMR analysis revealed that the reported morbidity rate for women (in the 20–59 age range) was much higher than the morbidity rate for men in both CD and UC cases. In addition, the CD:UC ratio, which has the advantage of normalizing for possible sampling biases, revealed a cluster of large values (i.e., relative risk of CD) in the North-West England which may require further investigation.

## Introduction

In humans, inflammatory bowel disease (IBD) is a collective term for the conditions of Crohn’s Disease (CD), Ulcerative Colitis (UC), Microscopic Colitis (MC) and Indeterminate Colitis (IC) characterized by chronic inflammation of the gastrointestinal (GI) tract [1–5]. CD and UC are the most common types of IBD. CD can affect any part of the GI tract, but typically affects the terminal ileum, colon and perianal area. It can occur in different phenotypic forms such as inflammatory, penetrating and stricturing, or different combinations of these forms [6–8]. UC, on the other hand, is marked by persistent and widespread inflammation of the colon, starting from the rectum and extending in a variable manner towards the caecum [9–11].

The incidence and prevalence of IBD are increasing worldwide [12–15]. Current estimates indicate that approximately 3.9 million females and 3 million males are affected by IBD globally [13]. Although the largest numbers of cases worldwide occur in the USA and China [15], the highest incidence rates have been reported in Canada [16,17], Northern Europe [6,18] and Australia [19]. Currently, approximately 0.2% of the European population is reported to have IBD [20]. Among European countries, the UK has one of the highest incidence and prevalence rates of IBD [6,20,21], including when age-standardized [13]. Studies have shown that the incidence and prevalence of IBD in the UK have risen steadily over the past few decades [22,23]. Currently, an estimated 540,000 people in the UK live with Crohn’s and Colitis (CCUK, 2024). It was reported that the prevalence of IBD is higher in countries with a high socio-demographic index (SDI) [13,15], such as the UK, and that the burden of IBD has consistently increased over time [13,14].

Some epidemiological studies have revealed that environmental factors may play an important role in the pathogenesis of IBD [24–34]. Smoking is an especially important risk factor for CD and increases the risk of disease recurrence and the need for surgical intervention. In addition, it was observed that the disease regresses positively when smoking is stopped [24]. Dietary practices have been related to the course of IBD, while the risk of IBD increases in diets high in saturated fatty acids and processed meats [25], it was observed that the risk of CD decreases in diets rich in fiber [26]. Further, it was found that the use of antibiotics increases the risk of IBD [27] and that the potential for developing CD may be higher when used at an early age [28]. Although environmental factors such as urbanization [29,30], air [31–33] and water pollution [34] have been studied as possible infection routes for IBD, little information is available about the etiology of the disease more generally.

The aim of this study was to provide a comprehensive and geographically explicit analysis of the spatial distribution of IBD cases, specifically CD and UC, across the UK, thereby filling a gap in the current literature by offering spatial insights that can aid in understanding the patterns of IBD prevalence and the possible role of environmental and geographical factors influencing these patterns. To achieve this aim, a new UK-wide IBD survey was conducted. Spatially referenced IBD data were collected through a UK-wide online survey (wp.lancs.ac.uk/ibdsurvey; ethically approved by Lancaster University), which included current postcode, postcode at first diagnosis and previous postcodes (up to 15 years) (see Supporting Information). Further data on the UK’s population for use in interpreting the survey data, including age and gender distribution, were obtained from the WorldPop database (hub.worldpop.org). Using the acquired survey data, the spatial distribution of CD and UC cases in the UK at 10 km^2^ grid resolution was examined, including when normalized to the UK population in those grid squares, and when normalized between diseases (i.e., CD:UC ratio) to mitigate against any possible reporting bias. The age- and sex-standardized morbidity ratios (ASMRs) of CD and UC cases were also analysed.

## Materials and methods

### Study site, online survey and data

This study was focused on the whole UK. The study data, including an online survey of people with IBD, UK population data including age and gender, and the spatial grid boundary definitions are described in following sections. The survey data were collected through an online portal prepared using Qualtrics (Qualtrics, Provo, UT) on a dedicated website (wp.lancs.ac.uk/ibdsurvey).

In national surveys of diseases, such as reported here, it is important to maximize the response rate, while at the same time ensuring an even spatial coverage in terms of the likelihood of response, to avoid unintended reporting bias. Therefore, participants from the UK were recruited through advertisements placed with a wide range of IBD organizations in the UK (CCUK, IBDUK, CICRA, Cure Crohn’s Colitis, Crohn’s MAP Vaccine, Guts UK and IBD Coach). The survey was promoted on both the official websites and social media accounts of these organizations for 307 days (01 December 2021–3 October 2022). Repeat requests for participation were sent via CCUK which represents the largest membership group with 47 local user networks covering the UK spatially. Additionally, to reach larger audiences and increase the number of survey participants, a promotional page was created on Facebook to advertise the study.

The specific data collected on each individual participant in the online survey comprised demographics (age, gender, ethnicity and occupation), type of diagnosis (CD, UC, Microscopic Colitis and Indeterminate Colitis), family IBD history, and postcodes from the current address, address at first diagnosis and previous addresses (up to 15 years) to allow spatial localization of the results. Ethnicity and occupation were not included in the present analysis. First diagnosis postcodes of people diagnosed with CD and UC were mapped in ArcGIS. Only the first diagnosis postcodes were included in the statistical analysis reported here, as these are most likely to reflect the geographical context in which the disease first emerged.

Ethical approval for the survey was granted by Lancaster University Faculty of Health and Medicine Ethics Committee (FHMREC20164). All participants under the age of 16 were requested to answer the questions with permission from their guardians. All participants from outside the UK and those submitting missing/incorrect/unlocalized information, were excluded from the final dataset.

### UK population data

Population data were required both to identify the general level of CD and UC risk at the grid level, and to analyse relative disease risks for different age and gender groups. For this purpose, data on the estimated total number of people (per approximately 100 m) were obtained from Worldpop.

### Spatial grid boundaries

Since the focus of this research was to examine the spatial distributions of CD and UC cases and CD and UC risks using the survey data, it was decided to conduct the analysis on units of pixels. Considering the UK-wide distribution of cases, pixel units can be useful for revealing regional differences and have the advantage of consistency in the sampling support (e.g., the size, geometry and orientation of the space on which the observations are defined). In this way, small but important focal areas can be identified, and a more detailed picture of the case density can be obtained. In this context, the UK geography was divided into 10 km^2^ grid squares in ArcGIS Pro 3.0. To determine the relative risk of CD and UC in each grid square, the ratio of the total number of reported CD and UC cases relative to the general population in the relevant grid squares was calculated.

### Age- and sex-standardized morbidity ratio (ASMR)

ASMR is a statistical measure that is used to compare the observed number of cases in a particular population to the expected number of cases in a standard population for a particular age and/or sex category. The ASMR allows comparison of disease incidence while accounting for age and sex distributions [35] and is described as:


$$\begin{array}{c} SMR=\frac{Observednum.ofcases}{Expectednum.ofcases} \end{array}$$ (1)

$$\begin{array}{c} Expectednum.ofcase=\frac{Observednum.ofcases}{UKpopulation}xpop.ofeachage\&gendergroup \end{array}$$ (2)

The observed number of cases is the total number of CD or UC positive cases reported in the UK-wide IBD study, while the expected number of cases represents the total number of observed cases divided by the total population, multiplied by the total population of each age and gender group. The age groups for both sex types were defined as 0–9, 10–19, 20–29, 30–39, 40–49, 50–59, 60–69 and 70 + .

An ASMR of < 1.0 means that there are fewer cases than expected in the local population. Conversely, an ASMR of > 1.0 indicates that the observed number of cases in the local population is higher than expected. In the case of equality, the number of observed cases equals the number of expected cases in the study population.

## Results

### Exploratory data analysis

The UK-wide IBD survey produced a total of 5,452 participants who reported four different IBD conditions: Crohn’s Disease (CD), Ulcerative Colitis (UC), Microscopic Colitis (MC) and Indeterminate Colitis (IC). The numbers of individuals identified for each IBD condition were as follows: CD (2,672; 49%), UC (1,946; 35.7%), MC (24; 0.44%) and IC (292; 5.35%). Participants from outside the UK, participants submitting missing/incorrect/unlocalized postcodes and those who did not answer the IBD and postcode questions were excluded from the study (518; 9.5%). Therefore, the final numbers of reported cases of CD, UC, MC and IC were 2,085 (53.64%), 1,559 (40.10%), 19 (0.48%) and 224 (5.76%), respectively.

### Spatial distribution of IBD cases across the UK

**Fig 1:**
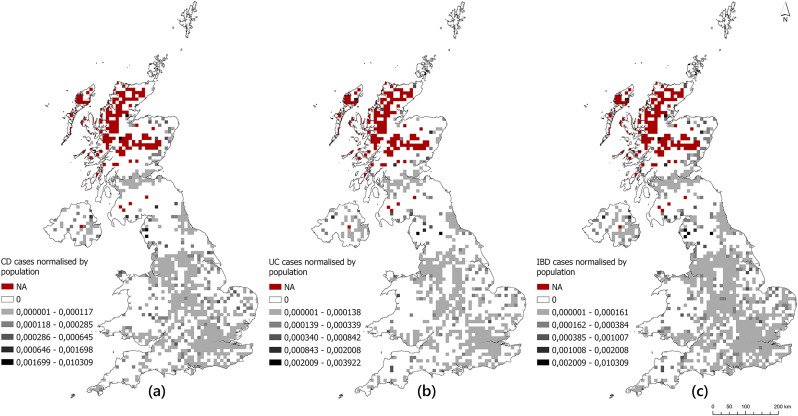
Distribution of IBD cases normalised by population across the UK according to the postcode at first diagnosis: (a) CD, (b) UC and (c) all IBD cases.

The location where a patient was first diagnosed was selected as defining ‘location’ for this study (postcodes post-first diagnosis were disregarded as they cannot influence onset). Maps of CD, UC and all IBD cases normalized by the population and spatially referenced to the postcode at first diagnosis are given in Fig 1.

Examining the distribution of reported IBD cases (participated in survey) normalised by population across the UK, that is, relative risk, it was observed that grid squares with no population (marked in red) are concentrated in Scotland. Grid squares with a positive population, but no survey participation (marked in white), are common for CD and UC in rural areas such as Wales, parts of Scotland, and the North, South-West and East of England. CD relative risk is higher in some grid squares in Wales, Northern England and Southern Scotland (Fig 1A), while UC relative risk is higher in a few grid squares in Northern England and Southern Scotland (Fig 1B). It was observed that risk is high for both CD and UC cases in some grid squares in Scotland, due to low sample size.

### Age and gender distribution across the UK

The age and gender of all IBD cases participating in the study were examined and grouped accordingly. For reported CD cases, the age range was 1–81 years. The age ranges of 1,529 female (73.3%) and 539 male (25.85%) CD cases were found to be 8–81 years and 1–79 years, respectively. In terms of gender, 5 participants (7–59 years) preferred not to answer, 6 participants (18–42 years) answered non-binary, 1 participant (37 years) answered trans-feminine, 1 participant (35 years) answered agender. Although three participants (33–64 years) chose the preference to self-identify, they did not provide any input in the relevant field.

The age range of the UC cases was found to be 6–95 years, while four UC participants answered the age question inconsistently. The age ranges of 1,196 female (76.71%) and 357 male (22.89%) UC cases were also found to be 7–95 years and 6–80 years, respectively. One participant (34 years) did not respond even though they preferred to choose to self-identify and one participant (40 years) answered non-binary.

The age range of IC cases was found to be 6–83 years, while one participant answered the age question inconsistently. The age ranges of 169 female (75.44%) and 53 male (23.66%) IC cases were 13–83 years and 6–79 years, respectively. One participant (26 years) answered non-binary. Of the cases included in the study, MC cases were the least reported of the IBD types. In total, all 19 MC cases were women, and the age range was 32–75 years.

**Fig 2:**
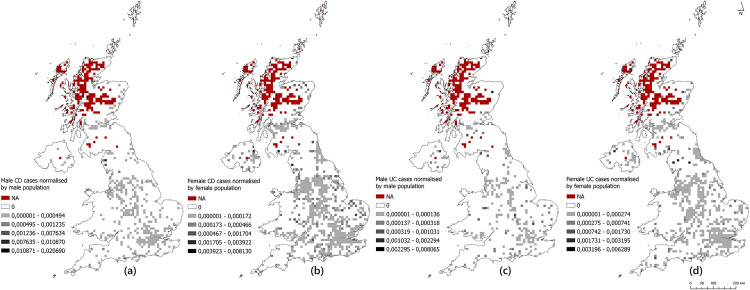
Distribution of (a) male and (b) female CD cases, and (c) male and (d) female UC cases, normalised by population across the UK.

The spatial distribution of CD and UC cases by gender, normalized by the population, is given in Fig 2. The reported relative risk appears to be clustered in densely populated urban areas, and it is seen that the female population, which constitutes nearly ¾ of the survey participants for both IBD types, has a more extensive distribution compared to the male population for both CD and UC (Fig 2).

### Age- and sex-standardized morbidity ratio

**Fig 3:**
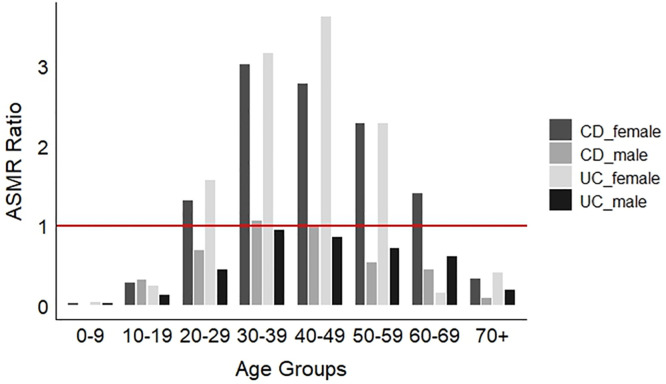
ASMR for CD and UC by gender plotted against age, represented in eight age categories. The horizontal red line represents equity of risk such that risks above the red line represent greater than expected risk and risks below the red line represent less than expected risk.

ASMR analysis results for both the CD and UC cases, calculated according to both age and sex, are given in Fig 3. Among male CD cases, an ASMR >1 was detected only for the 30–39 age group, while an ASMR<1 was observed for all other male age groups. For the female CD cases, an ASMR>1 covered a wide range between the ages of 20–69 and an ASMR<1 was found only in cases 0–19 and 70 + .

For the UC cases, no ASMR>1 value was detected in any of the age groups of male UC cases. For female UC cases, an ASMR>1 value was detected in the 20–59 age range, while an ASMR<1 was found only for the 0–19 and 60–70 + age categories.

Fig 3 shows that females between the age categories of 20–29 and 60–69 (CD) or 50–59 (UC) have significantly higher reported morbidity rates for both CD and UC than men, and this rate is particularly pronounced in the 30–39–50–59 age range. For the age categories 30–39 and 40–49, the ASMR for females is about three times higher than expected. This analysis, thus, shows that middle-aged women exhibit the highest reported morbidity rates for both CD and UC.

### Inter-disease risk analysis

**Fig 4:**
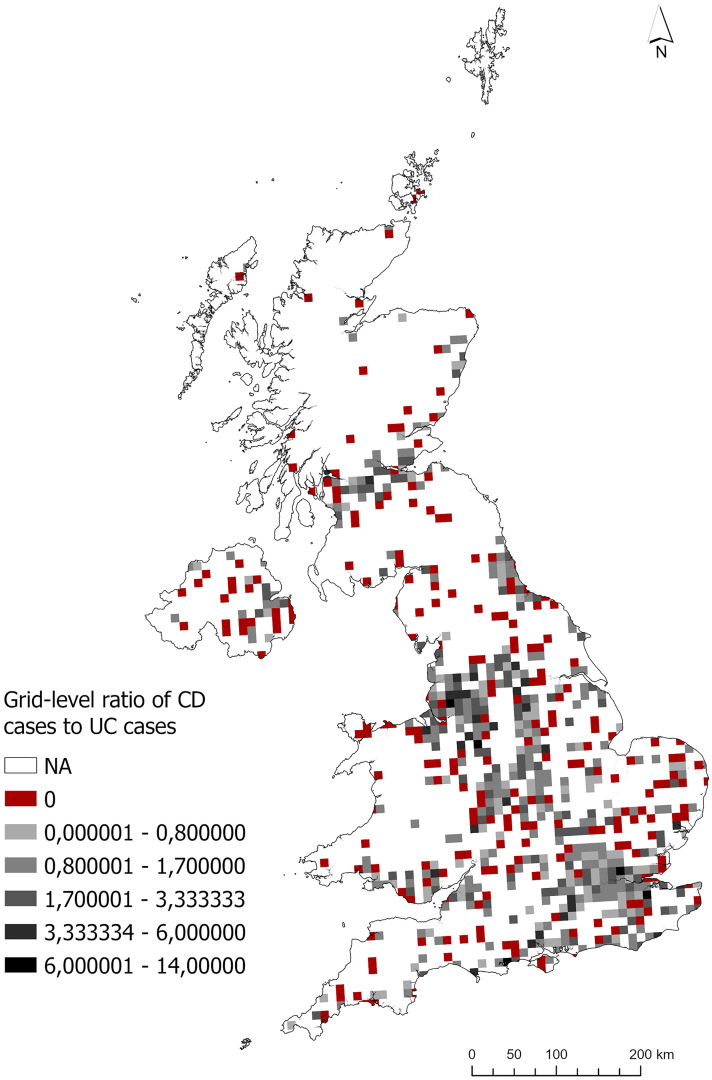
Distribution of CD:UC ratio for each grid square across the UK. White squares represent grid cells where calculation of a ratio is not possible (i.e., UC is zero), and red squares represent grid cells where the ratio is zero (i.e., CD is zero and UC is positive).

The number of reported CD and UC cases in each grid cell was used to calculate the CD:UC ratio and its distribution was mapped across the UK (Fig 4). In some grid squares, the number of UC cases was zero, while in some grid squares, both CD and UC cases were zero. These grid squares where the UC count was zero and calculation of a proportion was not possible were recorded as NA and marked in white in Fig 4. Cells where the CD count was zero, but the UC count was positive are marked in red.

It can be seen from Fig 4 that the CD:UC ratio was relatively high in some grid squares in North-West England around Manchester. Similarly high ratios can be seen in some isolated grid squares in Scotland (Edinburgh and Glasgow) and London. Lower CD:UC ratios can be seen in many grid squares across the UK and are more common in London and Birmingham. It is worth noting that there exists greater spatial variation in the CD:UC ratio across the UK than in the CD:population and UC:population ratios presented in Figs 1 and 2, which exhibited mostly small values with some isolated peaks.

## Discussion

### Discussion of results

The main aim of this study was to report the design, execution and results of a novel UK-wide survey on four diseases within the general IBD class. We used the resulting survey data to provide a preliminary investigation of the geographical distribution of CD and UC cases in the UK, calculated the ASMR of the CD and UC cases, and calculated relative IBD risk based on both the underlying population and by comparing the diseases to each other, with the latter having the benefit of mitigating against possible reporting bias. The data used consisted of the georeferenced cases for the four diseases obtained from the UK-wide IBD survey (wp.lancs.ac.uk/ibdsurvey), and population data including age and sex obtained from WorldPop (www.worldpop.org). Therefore, this study, carried out at a spatial resolution of 10 km^2^ grid squares, is novel in this context.

The data on the 5,452 cases who participated in the online survey, were classified according to IBD type, age, gender and address (current, first diagnosis and previous postcodes (up to 15 years). Due to an insufficient number of participants, the MC and IC cases were not considered in the analysis. For both the CD and UC reported cases, approximately ¾ of the participants for both types of IBD were female. The female:male ratios were found to be 2.8 and 3.3 for CD and UC cases, respectively. Brant and co-workers reported in their analysis of data from several large-scale studies that female CD cases outnumbered male CD cases, with a female: male ratio ranging from 1.2 to 2.2, but no significant difference was observed when UC cases were considered [36]. A study conducted in Western countries on the risk of developing IBD according to age found that women are more likely to be affected by CD after the age of 10 and that this risk peaks in their 70s; while men are less likely to develop UC before the age of 45, this risk increases after the age of 45 [37].

The UK has one of the highest incidence and prevalence rates of IBD in Europe [6,13,20,21]. In places where the incidence is high, CD cases are mostly seen in young women (20–40 years old), while they are seen in men at older ages [38]. The age-specific incidence of ulcerative colitis (UC) in women decreases with age [18] and many women have been reported to become infected with IBD during their reproductive years [39]. The risk of CD is lower in those under 20 years (up to 10–14 years) and this risk may increase in later years [37]. It was determined that the lowest age range in which women’s CD and UC morbidity is > 1 is 20–29 and the highest age range is 50–59, revealing strong demographic differences in IBD amongst women. It is unknown whether this reflects higher survey participation rates for women although the literature suggests that this might be the case [40–42].

Considering the distribution of CD and UC cases in the UK according to first diagnosis postcodes, the largest clusters were observed in core urban areas such as London, Birmingham, Manchester, Leeds and Scotland, as expected because participation is related to population density. However, considering the inter-disease risk analysis (CD:UC ratio), surveyed CD cases were higher than UC cases in the North-West England in the Manchester area. In contrast, large values were observed in London and Scotland in only isolated grid cells. Further analysis of possible environmental factors and exploration using different spatial resolutions (e.g., different grid cell sizes) may be useful in identifying risk factors around Manchester and may help to understand why the ratio of CD to UC cases is elevated there. While the CD:UC ratio provides a means of comparing disease types within the same sample and helps reduce the influence of population-related reporting bias, no additional statistical correction (e.g., smoothing or Bayesian adjustments) was applied to account for small counts. Therefore, the observed spatial patterns, particularly in grid cells with few cases, should be interpreted with caution.

Analysis of the ASMR for CD and UC revealed that the age range in which the ASMR value was greater than 1 in both men and women for both types of IBD was observed in the 30–39 age group. Especially in women, the ASMR value for both CD and UC was greater than 1 in the 20–59 age range, which reveals that young, middle-aged and older adult women may have a higher risk of contracting, developing and ultimately reporting both CD and UC. In addition to the finding that women, in particular, may face the risk of CD at an early age [37], we found a wide age range at which women may have an elevated risk compared to men, and this finding is supported by other studies [43].

### Limitations

We acknowledge several limitations of this study. These include reaching a small proportion of the likely population of IBD cases in the UK and possible misdiagnosis of IBD types. Since the number of reported cases constitutes the backbone of the study, we collected data through an online questionnaire with wide availability. Many voluntary charities and organizations were contacted and the UK-wide IBD survey was promoted periodically on social media and across several online platforms. We aimed to maximize our reach to as many IBD sufferers across the UK as possible and ensure an even geographical coverage, for example, by contacting potential respondents through CCUK, which has 47 local user networks covering the UK evenly. However, we acknowledge that it may not have been possible to reach cases who did not follow IBD organizations and/or who did not use social media. Furthermore, it is difficult to compare this survey with others as the data collected by others were for biomedical analysis (Nottingham/Astra ZenecA prospective IBD cohort study, 2022–2024) or more numerical, analysing for location (CCUK/Nottingham University Crohn’s and Colitis Survey, 2024).

A total of 3,085 grid cells were specified in the study, and the number of grid cells with no survey participation was found to be 2,276 (73.7%) for CD and 2,375 (76.9%) for UC. It was also determined that there was no population in 296 (9.5%) of the grid cells. Across the UK, an estimated 540,000 people live with Crohn’s Disease or Ulcerative Colitis. The total number of IBD cases included in the study is 5,452. Although this community participation survey represents one of the largest of its kind in the UK, the number of respondents, therefore, likely represents only 0.7% of all IBD patients, a small proportion of the overall case number in the UK [44]. To access more IBD case data in future studies, government health agencies could be contacted, or access to the online electronic general practitioner (GP) health records database could be requested [45]. A greater sample size would be useful in providing a clearer picture of local spatial distributions and clustering of IBD cases, and supporting analyses at the postcode level. This limitation should be considered when interpreting the spatial patterns observed in this study.

This study focused on regional variation in IBD prevalence, without exploring environmental determinants. We recognize that ecological factors, such as food environments, could play a significant role in the observed geographical distribution of IBD [46]. While our current study emphasizes the spatial patterns of CD and UC cases, we agree that a broader discussion of environmental factors, including diet, urbanization, and other ecological contributions, would strengthen the interpretation of our findings. In future work, we plan to incorporate a discussion on these environmental influences and refer to relevant studies that explore their potential impact on IBD prevalence.

The ASMR results may reflect, to some extent, a reporting bias in females related to males. The finding that the ASMR is highly consistent between CD and UC suggests that this may be the case, although further analysis is required to establish this more firmly. Thus, some caution must be exercised when interpreting these results. When diagnosing IBD subtypes, there is a possibility of misdiagnosis [47,48]. It is not always easy to distinguish CD from UC accurately, especially since they have similar symptoms, which leads to various difficulties in the diagnostic process. Data analysis was based on direct input from IBD survey participants and, therefore, input from clinically undiagnosed or misdiagnosed participants may be included. This may also relate to the consistency in some of the results for CD and UC, including in the ASMR analysis.

## Conclusions

This study presents a new survey and analysis of IBD cases in the UK and illustrates the spatial distribution and characteristics of the data acquired. Unlike previous studies that relied on survey-based case data analysis, our approach was novel in analyzing case numbers and population counts for each 10 km² cell across the UK. The maps of CD and UC relative risk (Fig 1) showed little spatial variation across the UK; some isolated peaks were observed in Scotland and a few other areas which may be related to small sample size. Interestingly, considering the CD:UC ratio, higher relative risk of CD was observed in core urban areas in the North-West of England, especially Manchester and Bolton, with lower risk in the periphery and rural areas. This finding will be explored in subsequent analysis. The ASMR analysis revealed that age-standardized risk of CD in the UK appears to be much greater in women than men and spread across a wide age range (ASMR = 1–3; ages 20–29–60–69). The results of this study may provide insights for understanding regional and demographic patterns of IBD incidence, particularly for Crohn’s Disease and Ulcerative Colitis. These findings could also serve as the basis for future research on factors influencing IBD risk. This analysis platform supports the spatial analysis of IBD cases by providing a scalable approach for grid-scale studies, particularly across multiple countries where larger datasets are available.

## Supporting information

S1 Data Age-gender, case, risk and population distribution data per grid. (XLSX)
